# A Rare Case of Submandibular Actinomycosis

**DOI:** 10.1007/s12070-023-03498-7

**Published:** 2023-02-24

**Authors:** Sanchit Bajpai, Navya Parvathareddy, Sweekritha N Bhat, Vijendra Shenoy S

**Affiliations:** Department of Otorhinolaryngology, Kasturba Medical College, Mangalore, Manipal Academy of Higher Education, Manipal, India

**Keywords:** Actinomycosis, Submandibular gland, Foreign body, Swelling

## Abstract

Actinomycosis is caused by actinomyces israelii, a gram positive nonacid fast, anaerobic, commensal bacterium within the oral cavity. Though it is a commensal in oral cavity, it seldom presents as cervicofacial actinomycosis. But there is limited access to literature proving cervicofacial actinomycosis existence so far. We report a case of 35 year old gentleman who presented with painless progressive neck swelling associated with pain while chewing food. On examination, 3.5*2.5 swelling was noted in right submandibular gland area, radiologically a foreign body was detected in the right submandibular gland. Mass was excised in toto and sent for HPE which revealed submandibular gland actinomycosis. Post operatively, patient was managed with long-term broad-spectrum beta lactams and was on regular follow-up. No evidence of recurrence/ fresh complaints noted till date. Although cervicofacial actinomycosis is rare and dangerous with debilitating complications, timely diagnosis and early intervention with broad spectrum antibiotics and surgical excision (if required) shows promising results with complete eradication of disease leaving no residues.

**Supplementary Information:**

The online version contains supplementary material available at 10.1007/s12070-023-03498-7.

## Introduction

Actinomycosis is caused by actinomyces Israeli, a gram-positive nonacid fast, anaerobic, commensal bacterium within the oral cavity [1]. It is not primarily limited to oral cavity alone; quite a few cases have been reported on cervicofacial actinomycosis [1], although there is not much literature supporting actinomycosis in the submandibular gland. We have encountered one such rare occurrence in our set up. Peculiarly our case is of a young gentleman without comorbidities or risk factors favoring growth of actinomyces. We report this rare case to bring awareness about this silent treacherous organism where early diagnosis and proper management gives positive results in almost all the cases with complete recovery.

## Case Report

**Fig. 1:**
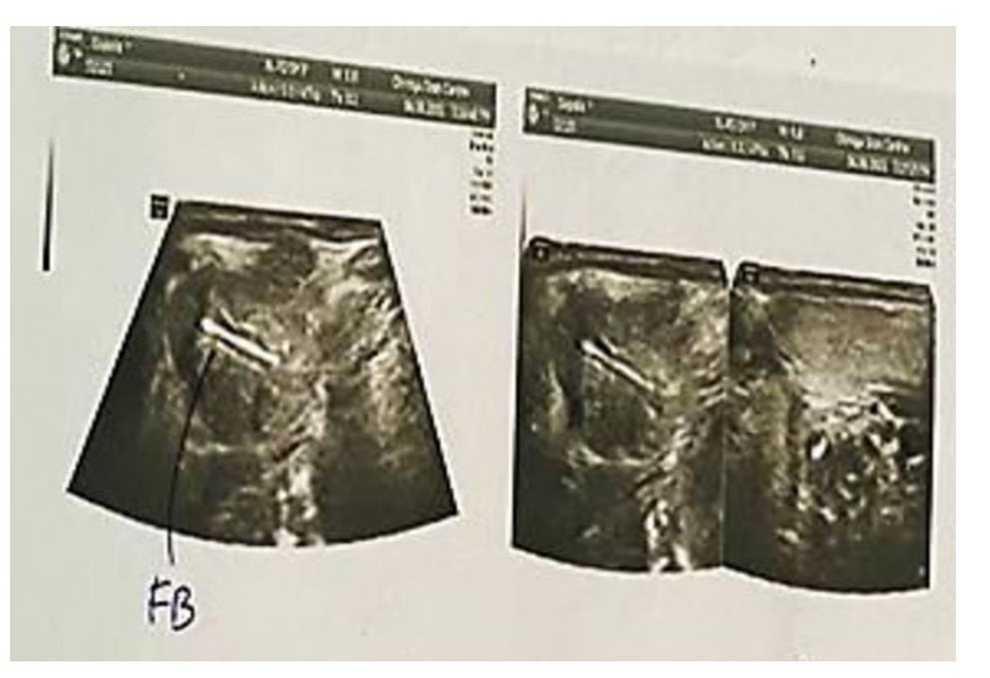
Ultrasound neck showing foreign body in right submandibular gland

**Fig. 2:**
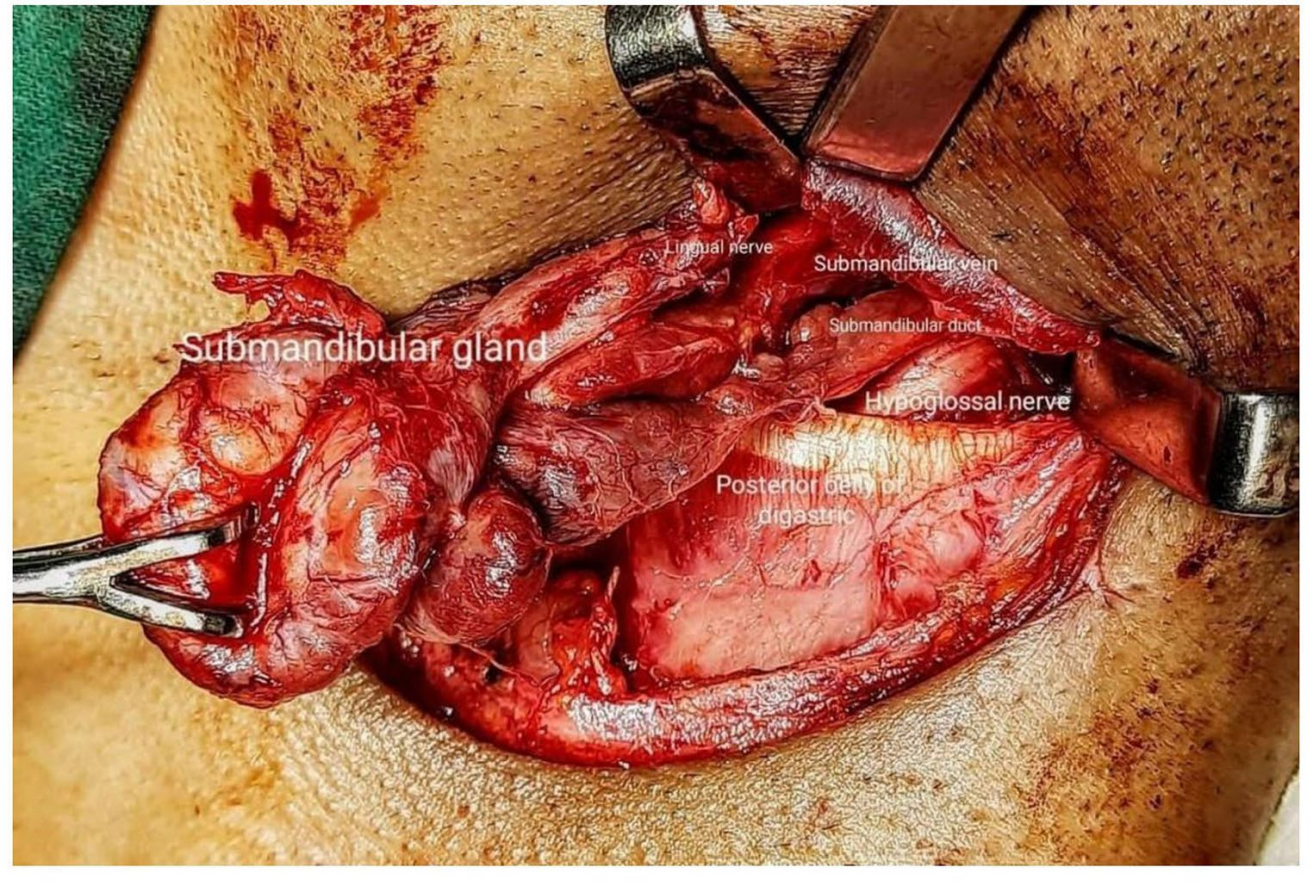
Intraoperative picture showing submandibular gland with structures in digastric fossa

**Fig. 3:**
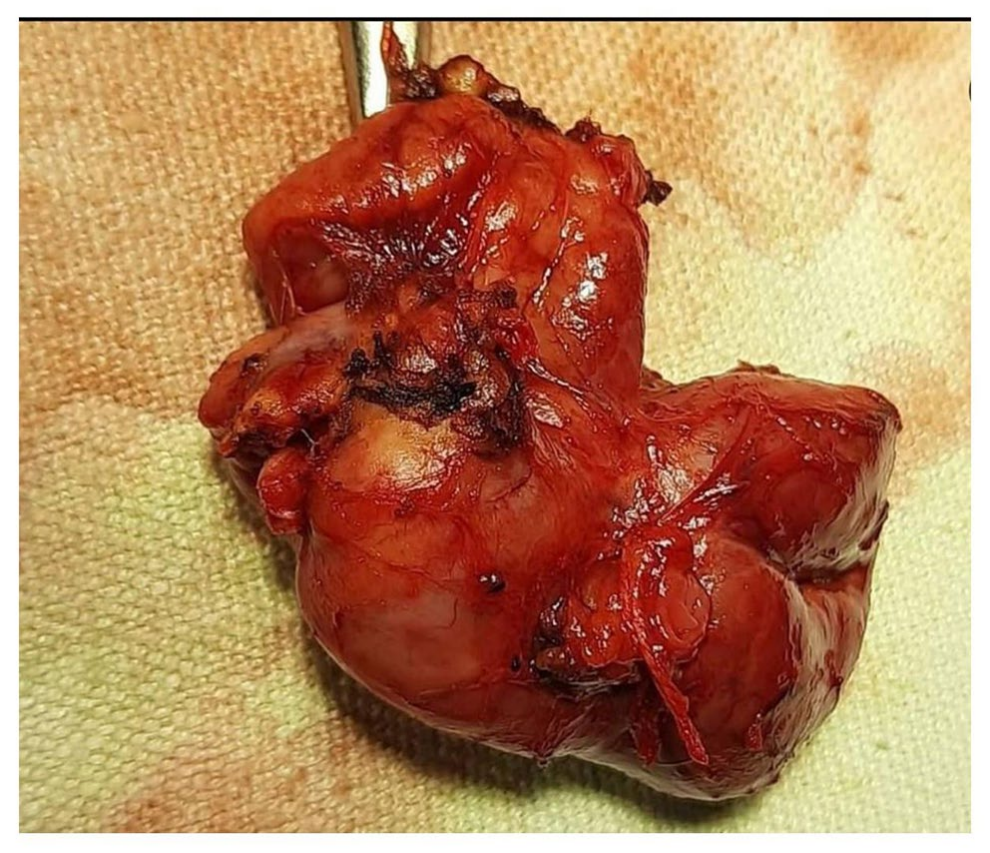
Gross specimen of submandibular gland having firm and rubbery consistency

**Fig. 4:**
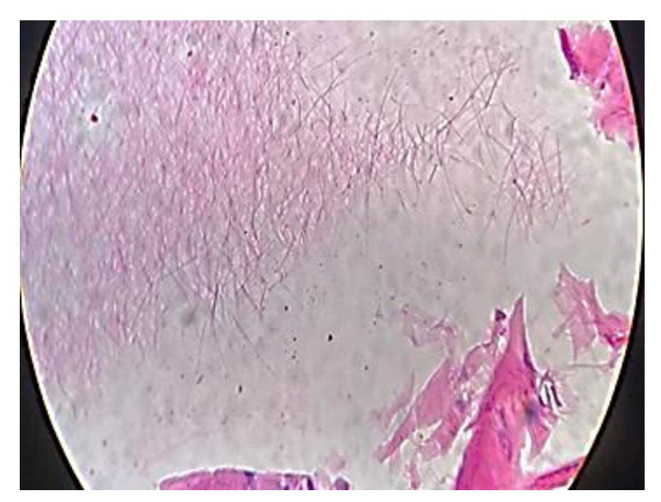
Showing characteristic clumps of basophilic filamentous bacteria in rosette like configuration

**Fig. 5:**
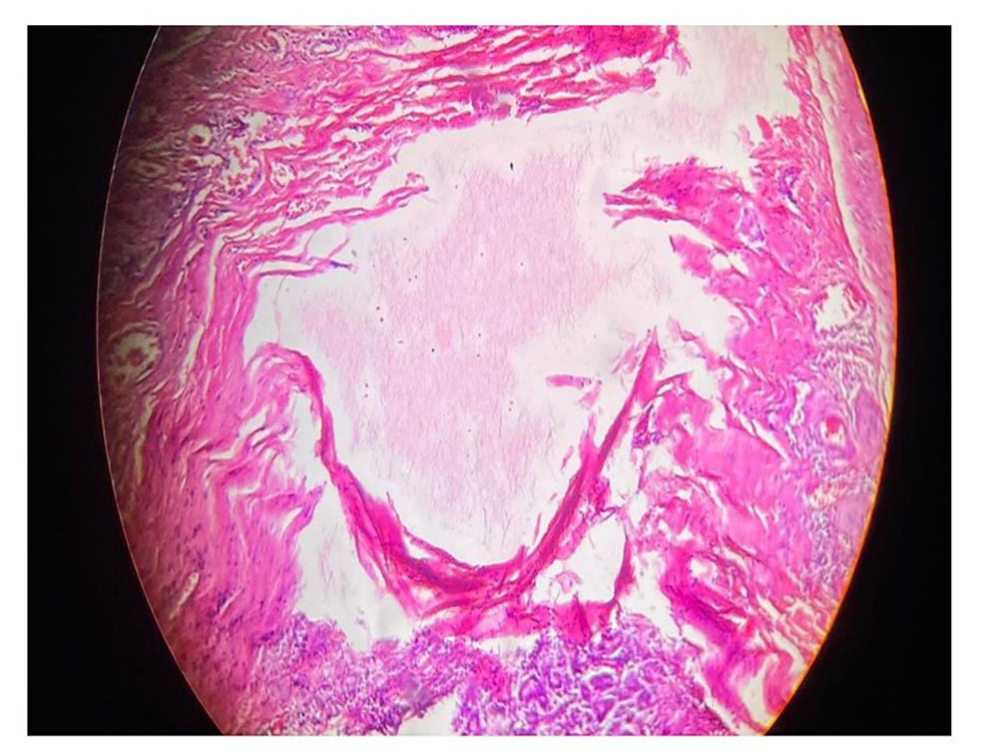
Granulomatous reaction with focus of actinomycial colonies with sulphur granules

A 35-year-old well-built gentleman presented to us with complaints of painless progressive swelling on the right side of neck for 1.5 months. He also had complaints of pain while chewing food for 15 days with no h/o difficulty in swallowing/hoarseness of voice/weight loss. Patient consulted local hospital for similar complaints one month back. An ultrasonography of neck was performed which revealed a foreign body within right submandibular gland as shown in Fig. 1; for which he was given a short course of antibiotics and analgesics which didn’t prove to give much benefit to the patient. Patient upon further questioning gave a history of accidental dry grass prick injury in the oral cavity while trying to clean his lower teeth a week ago, post that the site bled for a while and subsided spontaneously. On examination, 3.5*2.5 swelling was noted in right submandibular area, well defined, smooth surfaced, partially mobile, skin pinchable, non-tender, skin over swelling and surrounding skin normal with no discharging sinuses/ fistulas. Video laryngoscopy was done on the same day and was normal. The patient was counselled regarding the possible differential diagnosis including malignancy, and the need for right submandibular gland excision to confirm the diagnosis. The patient underwent right submandibular gland excision as shown in Figs. 2 and 3. Wound was closed in two layers and drain was placed in situ with adequate pressure dressing. Post-operative histopathological examination of the specimen revealed features of submandibular chronic sialadenitis with suppuration & actinomycosis as shown in Figs. 4 and 5. Based on the diagnosis made post operatively the patient was treated with intravenous crystalline penicillin 6 lakh units 6th hourly for the first 10 days. Regular dressing was done, and drain was removed on post op day 3. The patient was discharged in a stable condition on day seven on amoxicillin and clavulanic acid combination for 3 months. Patient was followed up every fortnight for the next 3 months. Until the patients last visit, there were no complaints or signs of recurrence of disease. Patient was advised to continue follow up for another 4 months (once a month). So far, the result of this patient is promising to label it as a successful treatment.

## Discussion

Actinomycosis is an opportunistic infection of oral cavity most common sites being tonsils & carious teeth. Three major areas where actinomycosis can occur are Cervicofacial, Pulmonary and Gastrointestinal. Cervicofacial actinomycosis has been reported quite a few times in literature [1], however encountering actinomycosis in submandibular gland is an extremely rare phenomenon. It usually presents with no predisposing factors, but few cases have been reported with poor oral hygiene, immunosuppression due to long term steroids and diabetes mellitus. Actinomyces is a part of normal flora of oral cavity, which can penetrate surrounding normal soft tissues, which explains its occurrence and spread in cervicofacial area [2].

Injury to healthy tissue has been mentioned as one of the predisposing factors for this rare condition to occur which was also seen in our case. Usually, actinomycosis cases present with discharging sinuses, hyperemia, febrile, tender mass [2]. To our surprise none of these features were noted, which drew a line towards malignant etiology bracing towards a painless progressive swelling within a short span.

Radiologically ultrasound fails to differentiate it from malignancy. However, contrast enhanced CT might help us bring close to diagnosis [3]. This disease is often characterized by abscess formation with surrounding granulomatous inflammatory exudates.

The characteristic finding of this infection is presence of ‘Sulphur granules’ from the aspirate. Patients usually present with abscess leading to discharging sinuses and fistulas. Although cervicofacial actinomycosis is infrequent, it is always ideal to rule this out from other pathologies like malignant or benign tumors to various granulomatous and mycotic infections [4].

The interesting aspect we noticed was the presence of a foreign body (grass root) within the gland substance on ultrasonography, which the patient had used frequently before for tooth cleaning. The patient presented with symptoms of swelling post injury. A possible explanation for its occurrence could be that the root of grass accidentally injured the mucosa of floor of mouth and gained its entry via the duct into the gland substance cultivating later into a florid suppuration. Foreign body induced reaction and suppuration with actinomycosis is itself an extremely rare phenomenon in submandibular region and has not been reported till date.

Medical management includes penicillin as treatment of choice [4, 5] for a period of 6 months to 1 one year, as actinomyces species are sensitive to beta-lactams. Although macrolides, tetracyclines, cephalosporins also can be given, not much benefit is noted. Keeping in mind the indolence of microorganisms, it is better to continue antibiotic course for an ample amount of time and not stop it prematurely. A study by Moghimi et al. stated that a surgical approach in combination with intravenous penicillin and metronidazole until clinical improvement is seen, followed by oral antibiotics for 2–4 weeks is generally efficient with better outcomes [5].

Bone permeation has also been noted in few cases [6] and is always associated with increased complications, so it is always ideal to prefer surgical management followed by treating medically with penicillin for a longer course. Literature so far stated that surgical debridement with shorter course of antibiotics would suffice. So, this case forms a base for a new treatment regime which improves success rate in treating this deadly disease and improves quality of life of the patient.

## Conclusion

Actinomycosis of submandibular gland is an extremely rare disease and often forms a diagnosis of exclusion. The unusual presentation as seen in our case creates a diagnostic dilemma and leads to treatment delay. Although cervicofacial actinomycosis is rare, dangerous with debilitating complications, thus early diagnosis and prompt treatment with surgical excision followed by long course of broad-spectrum antibiotics shows promising results with complete eradication of disease leaving no residues.

## Electronic Supplementary Material

Below is the link to the electronic supplementary material.

Supplementary Material 1
