# Supplementary material for: A Rare Case of Submandibular Actinomycosis
Source: Indian J Otolaryngol Head Neck Surg. 2023 Feb 24;75(3):2289–92. doi: 10.1007/s12070-023-03498-7 (PMC10447759; doi:10.1007/s12070-023-03498-7)
Supplement: Supplementary file 1 — Supplementary Material 1 [file 12070_2023_3498_MOESM1_ESM.doc]

From

# **Dr. Vijendra Shenoy S**

Department of Otorhinolaryngology,

Kasturba Medical College, Mangalore

Manipal Academy of Higher Education

Karnataka State, India

# To

Editor

**Indian Journal of Otolaryngology and Head & Neck Surgery**

**Dear Sir,**

I am herewith enclosing the manuscript **‘SUBMANDIBULAR ACTINOMYCOSIS: A RARE ENTITY’** for publishing as a case report in your journal. Each of the authors has contributed to, read and approved this manuscript. None of the authors has any conflict of interest, financial or otherwise. This manuscript is original and it, or any part of it, has not been previously published; nor is it under consideration for publication elsewhere.

Thank you.

**Regards**

**Dr. Vijendra Shenoy S**
